# Molecular Cloning, Expression and Characterization of Plasmid Encoding Rhomboid 4 (ROM4) of Tachyzoite of *Toxoplasma gondii* RH Strain

**Published:** 2017

**Authors:** Mohammad Taghi RAHIMI, Shahabeddin SARVI, Mahdi SHARIF, Saeid ABEDIANKENARI, Ehsan AHMADPOUR, Reza VALADAN, Ahmad DARYANI

**Affiliations:** 1. School of Medicine, Shahroud University of Medical Sciences, Shahroud, Iran; 2. Toxoplasmosis Research Center (TRC), Mazandaran University of Medical Sciences, Sari, Iran; 3. Dept. of Parasitology and Mycology, School of Medicine, Mazandaran University of Medical Science, Sari, Iran; 4. Immunology Department, Faculty of Medicine, Mazandaran University of Medical Sciences, Sari, Iran; 5. Infectious and Tropical Diseases Research Center, Tabriz University of Medical Sciences, Tabriz, Iran; 6. Molecular and Cell Biology Research Center, School of Medicine, Mazandaran University of Medical Sciences, Sari, Iran

**Keywords:** Apicomplexa, *Toxoplasma gondi*, *Rhomboid 4*, Cloning, Expression

## Abstract

**Background:**

The objective of this study was to clone, express and characterize the gene encoding rhomboid 4 (*ROM4*) proteins, a vital gene in surface adhesion and host cell invasion process of tachyzoite of *T. gondii* in an appropriate expression vector and eukaryotic cell for production of recombinant protein.

**Methods:**

*Toxoplasma* RNA was isolated from tachyzoites (RH strain) and complementary DNA was synthesized. Oligonucleotide primer pair was designed based on *Toxoplasma ROM4* gene sequence with *Xho*I and *Eco*RI restriction sites at 5′ end of forward and reverse primers, respectively. *ROM4* gene was amplified by PCR, cloned into pTG19-T vector and the recombinant plasmid was sequenced. The gene was subcloned into pcDNA3 plasmid and expressed in CHO cells as eukaryotic cell. SDS-PAGE and western blotting were performed for protein determination and verification.

**Results:**

Cloning of *ROM4* gene in pTG19-T vector was confirmed by colony-PCR and enzymatic digestion. The results of enzymatic digestion and gene sequencing confirmed successful cloning and subcloning procedures. The nucleotide sequence of the cloned *ROM4* gene showed 99% homology compared to the corresponding sequences of original gene. SDS-PAGE and western blotting analyses of the purified protein revealed a single band having expected size of 65 kDa.

**Conclusion:**

This eukaryotic expression system is an appropriate system for high-level recombinant protein production of *ROM4* gene from *T. gondii* tachyzoites used as antigenic component for serological assay and vaccine development.

## Introduction

*Toxoplasma gondii* is an obligate intracellular protozoan, which has worldwide distribution infecting humans and a wide range of animals (1). Seroprevalence of *Toxoplasma* infection widely varies between 30% and 60% in developed and developing countries (2, 3). *Toxoplasma* infection is considered as one of the most important public health problems. Felines are the final hosts playing a prominent role in epidemiology of this zoonotic disease by excreting and spreading oocysts of the parasite. The intermediate hosts particularly humans usually acquire the infection via accidental ingestion of contaminated food, water, or soil contaminated with oocysts and eating raw meat that contains tissue cysts of the parasite (4, 5).

Even though the majority of cases of *Toxoplasma* infection in healthy individuals are generally subclinical and asymptomatic, exposure to *T. gondii* during pregnancy can lead to vertical transmission to the embryo resulting in serious pathological complications including microcephaly, hydrocephalus, blindness, spontaneous abortion, and fetal death (6). Considering the increasing number of immunocompromised individuals such as HIV positive, organ transplant recipients and cancer patients, *T. gondii* as an opportunistic agent can lead to life-threatening disease for the mentioned groups (5, 7).

Owing to the significance of *T. gondii* in above-mentioned high-risk groups that lead to serious health problems as well as mortality in domestic animals, many efforts have been conducted on variant antigens of *T. gondii* in order to develop an effective vaccine against toxoplasmosis. So far, different antigens of *T. gondii* were known including Surface Antigen (SAG), Roptery Proteins (ROP), Dense Granule Antigen (GRA), Microneme Proteins (MIC), Rhomboid Proteases (ROMs), etc. (2, 8). Among them, rhomboids are ubiquitous family of polytopic intramembrane serine proteases cleaving their substrates within the transmembrane domain. The genomes of apicomplexan protozoa encode multiple rhomboids. Tg*ROM4* is important rhomboid of *Toxoplasma* tachyzoite involved during surface adhesion process and host cell invasion. In addition, Tg*ROM4* plays a vital role in parasite replication by cleaving Apical Membrane Antigen-1 (TgAMA1) to commence replication followed by invasion (9, 10). Gene encoding protein *ROM4* can be considered as a proper target for DNA vaccine and design diagnostic kits.

Therefore, the objective of the current study was to clone, express and characterize the gene encoding *ROM4* protein of RH strain of *T. gondii* in an appropriate expression vector and eukaryotic cell.

## Materials and Methods

### Preparation and proliferation of T. gondii tachyzoites

A highly virulent strain of RH *T. gondii* was kindly provided by the Toxoplasmosis Research Center (TRC) in Mazandaran University of Medical Sciences (Sari, Iran). To proliferate and collect tachyzoites were maintained in BALB/c mice by serial intraperitoneal passage of 1×10^6^ tachyzoites.

### Total RNA extraction

The obtained tachyzoites were washed twice with 10 mM phosphate-buffered saline (PBS), pH 7.4. Total RNA was extracted from tachyzoites using the RNX Plus kit (Cinnagene ® Cat. no.: RN7713C, Iran) according to the manufacturer’s instructions. RNA quality and quantity were evaluated by agarose gel electrophoresis and spectrophotometry, respectively.

### PCR amplification of ROM4 gene

Reverse transcription PCR (RT-PCR) was performed using complementary DNA (cDNA) Synthesis Kit (Fermentase ®, USA) in order to construct cDNA from the extracted RNA according to the manufacturer’s protocol. The following specific primers were used for amplification of *ROM4* gene using cDNA as the template: F: AAAGAATTCATGGTGTGGACTTCGGCCGTCG (forward primer, introduced *Eco*RI recognition site, underlined) and R: AAACTCGAGTTACGGTTCAAGATAATACTGC (reverse primer, introduced *Xho*Ι recognition site, underlined). The following PCR amplification conditions were included: initial denaturation at 94 °C for 7 min, then 35 cycles of denaturation at 94 °C for 1 min, followed by annealing at 62 °C for 40 sec, extension at 72 °C for 120 sec, and final extension at 72 °C for 10 min in a thermal cycler (BioRad C1000). The PCR product was analyzed by electrophoresis on a 0.7% agarose.

### Construction of recombinant vector (pTG19-ROM4)

PCR product was purified using DNA Extraction Kit from agarose gel (AccuPrep® Gel Purification Kit, Bioneer, South Korea), following the manufacturer’s instructions. The purified product was ligated into pTG19 plasmid vector (Sinaclon, Cat. no.: PR911643, Iran) according to the manufacturer’s instructions. The ligated plasmid was transformed to the competent bacteria, *Escherichia coli* (*E. coli*) Top 10 strain according to the protocol (10), and incubated in Luria-Bertani (LB) broth medium free antibiotic for 1h, at 37 °C. The transformed bacteria were cultivated on the LB agar plate containing ampicillin 100 mg/ml, Isopropylthio-β-D-galactoside (IPTG) 200 mg/ml, 5-bromo-4-chloro-3-indolyl-a-D-galactoside (X-Gal) 20 mg/ml and incubated overnight at 37 °C. To confirm the gene cloning, blue (non-recombinant) and white colony (recombinant and successful ligation) screening, colony-PCR method, enzymatic digestion, and sequencing were used. The plasmid was extracted from the bacteria (white colony) by plasmid extraction kit (Accu-Prep Plasmid MiniPrep DNA Extraction Kit, Bioneer®, South Korea), and digested by *Eco*RΙ and *Xho*Ι enzymes. The enzymatic reaction was performed in two conditions, single and double digestion, separately. These reactions were incubated for 16 h at 37 °C. The products were analyzed by electrophoresis on 0.7% agarose gel and the size of them compared with 1kb DNA ladder (GeneRuler ™ 1kb, Fermentase®, USA). The recombinant plasmid (PTROM4) was sequenced by Bioneer Corporation, South Korea and compared with previous records in gene bank.

### Construction of expression recombinant vector (pcROM4)

The recombinant plasmid and pcDNA3 plasmid (Invitrogen, Carlsbad, CA, USA) as an expression vector were digested with *Eco*RI and *Xho*I enzymes. The digested pcDNA3 plasmid and *ROM4* fragment were gel purified using DNA Extraction Kit (AccuPrep® Gel Purification Kit, Bioneer, South Korea). The *ROM4* fragment was ligated into the digested pcDNA3 as described previously (11). The ligation product was transformed into the *E. coli* Top10 strain, according to the standard protocol and cultured in Luria-Bertani (LB) broth medium free antibiotic by incubating for 1hr at 37 °C with shaking. The transformed bacteria were plated onto LB agar plates containing ampicillin 100 mg/ml and incubated at 37 °C for 16–18 h. To confirm the pc*ROM4* recombinant plasmid, Colony-PCR amplification and restricted digestion with the *Eco*RI and *Xho*I enzymes were used and the recombinant pc*ROM4* plasmids were sequenced using T7 promoter and BGH-rev (universal primers) by Bioneer Company.

### Expression of recombinant ROM4 protein in CHO cells

The Chinese Hamster Ovary (CHO) as a eukaryote cell was cultured under sterile conditions in Roswell Park Memorial Institute medium (RPMI) with 10% FCS at 37 °C with a 5% CO2 conditions. Pc*ROM4* was transfected into CHO cell using TurboFect Transfection Reagents (Fermentase ®, USA) using 3:1 and 6:1 ratio transfection reagent to plasmid. Transfected cells were seeded in 12-well culture plates with DMEM containing G418 (Santa Cruz, USA) (400 μg/ml) and kept at 37 °C with a 5% CO2 conditions for 14 d. Appropriate controls including un-transfected CHO cells and CHO cells transfected with empty pcDNA3 were used in this study. Then, pc*ROM4* expression in transfected cells was evaluated by RT-PCR, sodium dodecyl sulfatepolyacrylamide gel electrophoresis (SDS-PAGE) and western blotting analysis (WB).

### RNA extraction and RT-PCR procedure

The transfected and un-transfected cells were collected from the media and washed with ice-cold PBS for three times. To evaluate the expression of the recombinant plasmid in CHO cells at the mRNA level, RT-PCR was used. Total RNA was extracted from the collected cells by RNX (Plus) RNA Isolation Kit and RT-PCR were carried out using RT-PCR kit (Fermentase ®, USA) according to the manufacturer’s instructions.

### SDS PAGE and WB analysis

The transfected and un-transfected cells were rinsed with ice-cold PBS and harvested on ice with 200 μL RIPA lysis buffer (50 mM Tris pH 7.4, 150 mM NaCl, 1% Triton X-100, 1% Sodium deoxycholate, 0.1% SDS) containing protease inhibitor cocktail (p2714, Sigma, USA). The cells were then exposed to freeze-thaw cycles. Then the cell lysates were boiled in the loading buffer for 10 min and separated using a 10% (w/v) Gel Electrophoresis (SDS-PAGE) according to the standard protocol. Following electrophoresis, the separated proteins were transferred to the 0.2 μm nitrocellu-lose membrane (Sigma-Aldrich) using a Mini trans-blot electrophoretic transfer cell (Bio-Rad, USA) in transfer buffer containing 25 mM Tris (pH=8.3), 192 mM glycine and 20% methanol at 80v for 1 h. The membrane was blocked with 2% Bovine Serum Albumin (BSA) diluted in Tris-buffered saline with Tween 20 (PBST) (0.5 M NaCl, 0.02 M Tris pH = 8.5, 0.05% Tween 20) for 16–18h at room temperature then washed three times with TBST. The membrane was incubated with 1:500 dilution seropositive mouse antibodies for 2 h at 37 °C, followed by washing as previously described. The membrane was incubated with 1:3000 diluted goat anti-mouse IgG Horseradish Peroxidase (HRP) secondary antibodies for 1 h and washed. The page was developed using chromogenic substrate Di Amino Benzidine (DAB)/ H2O2 substrate solution for 15 min at room temperature. The reaction was stopped by washing three times in distilled water.

## Results

**Fig. 1:**
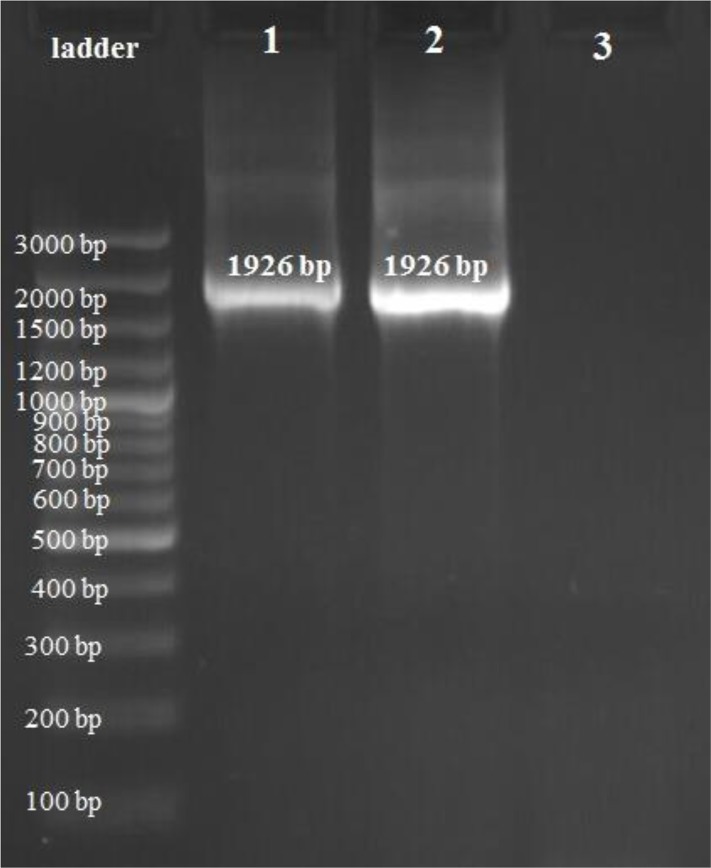
Gel electrophoresis analysis on PCR product, Lane 1&2: *ROM4* gene with the expected band size. Lane 3: negative control

**Fig. 2:**
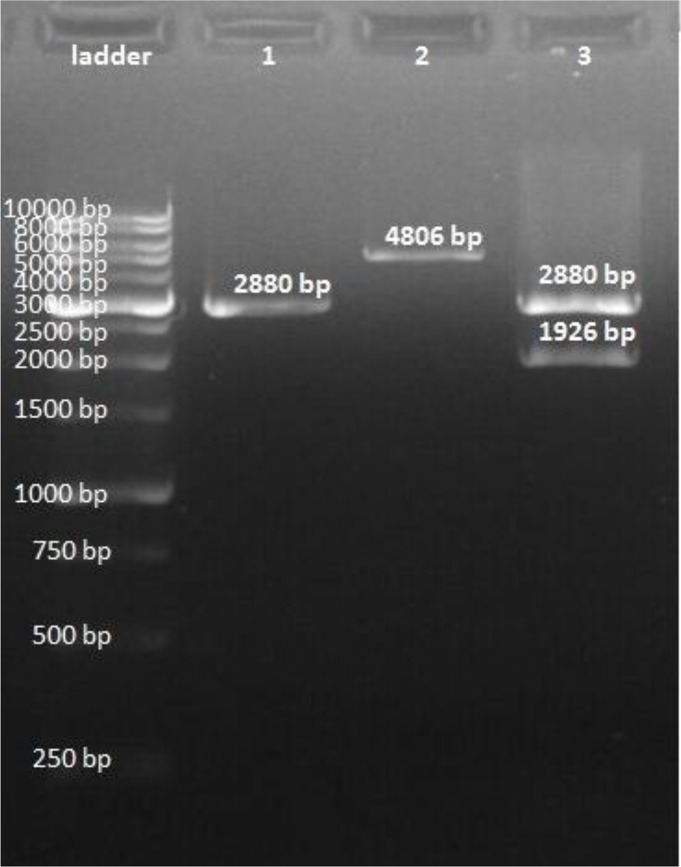
Digestion of pTG19T vector containing *ROM4* fragment by EcoRI and XhoI restriction enzymes. Lane 1: pTG19T vector without insert, Lane 2: single digestion, Lane 3: double digestion

Total RNA was extracted from tachyzoite of *T. gondii* RH strain and evaluated by agarose gel electrophoresis. The extracted RNA was used as the primary source to produce cDNA by reverse transcription PCR (RT-PCR). The PCR products were analyzed on 0.7% (w /v) agarose gel and a single band with the correct size (1926 bp) pertaining to the amplification of *ROM4* gene was observed (Fig. 1). Cloning of *ROM4* gene in pTG19 vector was confirmed by observing both white (recombinant) and blue (non-recombinant) colonies appeared on ampicillin-LB agar. The recombinant plasmid pTG-*ROM4* was successfully digested with *Eco*RI and *Xho*I restriction enzymes (Fig. 2). The recombinant plasmid of *ROM4* was sequenced and the sequences were submitted to GenBank under the following accession no.: KT715444. The nucleotide sequence of the cloned *ROM4* gene showed 99% homology compared to the corresponding sequences of the original gene in GenBank (accession no.: GbAY596193).

**Fig. 3:**
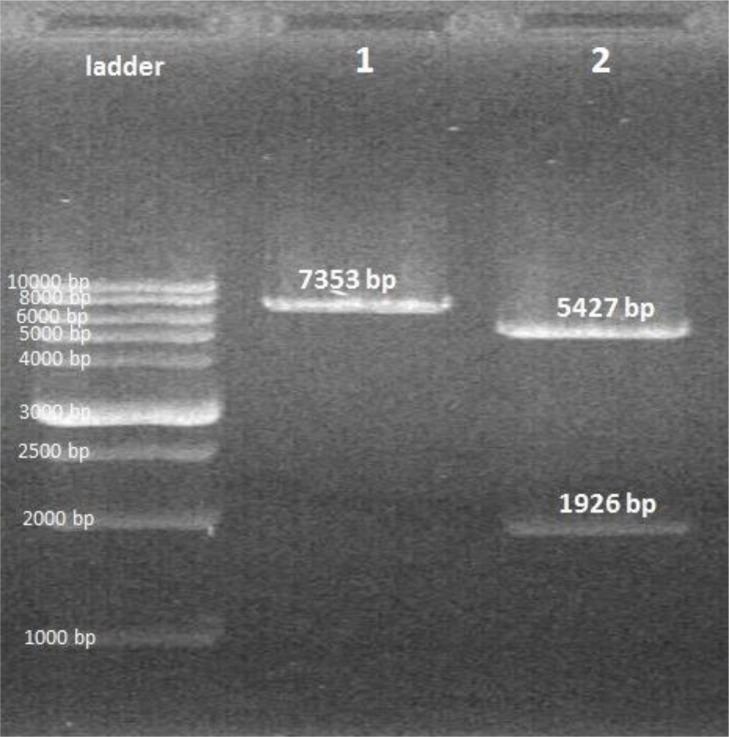
Digestion of pcDNA3 vector containing *ROM4* fragment by EcoRI and XhoI restriction enzymes. Lane 1: single digestion, Lane 2: double digestion

The digested band (*ROM4* gene) was extracted and subcloned into pcDNA3 expression vector. After transformation of the expression recombinant vector to *E. coli* Top10, analysis of the recombinant plasmids using colony PCR, restriction enzyme (Fig. 3) and sequencing confirmed that ROM fragment successfully was subcloned into pcDNA3.

**Fig. 4:**
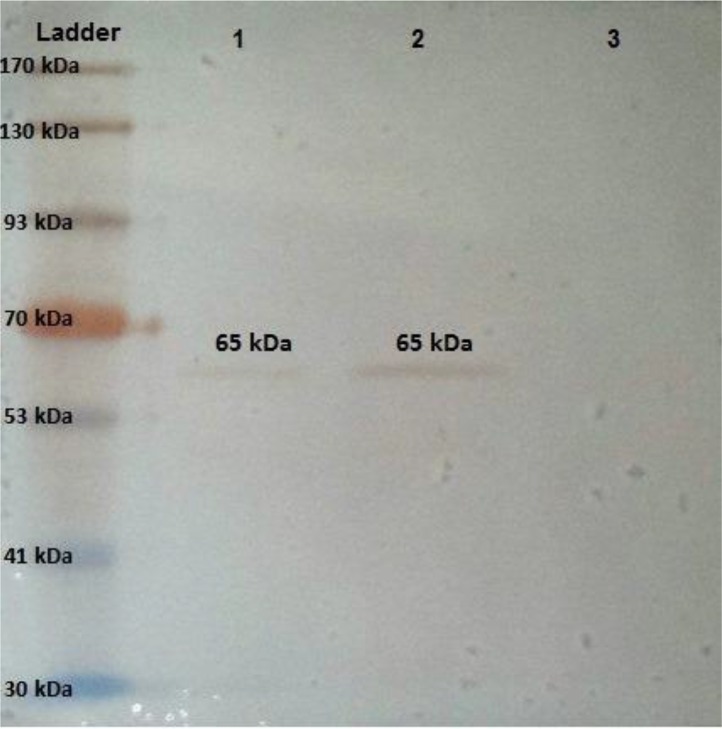
WB analysis of recombinant pcROM4. Lane 1&2: transfected cells containing pc*ROM4* plasmid with 3:1 and 6:1 ratio of transfection reagent to plasmid, respectively. Lane 3: pcDNA3 without *ROM4* fragment as a negative control

Then, pcROM4 was transfected in CHO cells and *ROM4* protein was expressed by the eukaryotic cells. SDS-PAGE and WB analyses of the purified protein showed a single band having expected size of 65KDa (Fig. 4).

## Discussion

There is no effective vaccine in humans and Toxovax (S48 strain) is the only accessible commercial vaccine in sheep licensed in Europe and New Zealand but there is safety concern due to recrudescence of the infection and inadequate efficacy (12). In addition, drug treatment of the infection just can control the proliferative tachyzoite stage causing acute phase of the disease in human but cannot eliminate infection, especially for tissue cysts of *T. gondii* (13). Vaccination against toxoplasmosis also would have economic advantages, as it would decrease the financial burdens of lifelong care required for individuals with severe chronic disease. The ideal vaccine to use in veterinary field would have not only benefits of increasing livestock productivity but also decreasing the public health risk contributed to eating meat products with tissue cysts (5). Therefore, the development of an efficient and safe prophylactic vaccine inducing protective immunity in humans and animals against toxoplasmosis is urgently needed. *ROM4* plays a prominent and major role in the process of surface adhesion and facilitates host cell invasion of the parasite*.* Therefore, gene encoding protein *ROM4* can be leading candidate and proper target for development of an effective vaccine against toxoplasmosis (10). The entire gene encoding *ROM4* is 1926 bp in length.

In the current study, sequencing of our gene showed 99% identification to the registered *T. gondii ROM4* gene sequence in Gen-Bank and *ROM4* gene was successfully cloned into pTG19-T vector and into pcDNA3 expression vector for protein expression. *T. gondii* contains six rhomboids expressed differentially during life cycle of parasite that localize to different successive compartments along the secretory pathway. ROM1, ROM4, and ROM5 being expressed in tachyzoites stage while ROM2 and ROM3 are present in sporozoites and ROM6 seem to be mitochondrial PARL-like rhomboids (14–16).

DNA fragment of *T. gondii* ROM1 gene was amplified and inserted into pMD18-T vector to construct a recombinant plasmid pMDROM1. TgROM1 open reading frame fragment was subcloned into eukaryotic expression vector pVAX1 to form pVAX-ROM1 through *Eco*RI and *Hind*III sites. Recombinant plasmid pVAX-ROM1 was transfected into Hela cells (17). Micronemes (MIC2, MIC3, and MIC4) are important antigens of tachyzoites of *T. gondii* for both binding to the host cells and invasion of the organism (18). MIC2 protein complex is a major virulence determinant for *Toxoplasma* infection and that MIC2-deficient parasites constitute an effective live-attenuated vaccine for experimental toxoplasmosis. This important and vital function of microneme depends on *ROM4* and it is not possible without presence of ROM4. Indeed, this antigen affects the processing of surface adhesions and facilitates host cell invasion of tachyzoites*.* Therefore, *ROM4* is a trigger for microneme (19).

A study amplified MIC1 in *T. gondii* ZS2 isolate and the gene was inserted to pWR450-1 by digesting with restriction enzymes and linking reaction (20). The gene fragment coding MIC1 from the genomic DNA of *T. gondii* was amplified, inserted into pGEM-T and transformed into *E. coli* XL-1 by heat shock method and analyzed by digestion using restriction enzymes including *Pst* I, *Hind* III, *Nco* I and *Eco* RV. Analysis of gene sequence indicated 97%–98% homology with gene encoding MIC3 protein of tachyzoite RH isolate (21). An investigation amplified cDNA of GRA8 from tachyzoite of *T. gondii* RH strain and cloned in pET−28b (+) and expression of recombinant GRA8 was evaluated by SDS-PAGE and western blotting. The cloned gene fragment had complete homology with the published sequence of GRA8 gene in Gen-Bank by sequence analysis. The expected molecular weight of native GRA8 (35 kDa) was detected (22). GRA5 gene was amplified, cloned in pcDNA3 and transfected in CHO cells for analysis of expression of GA5 protein. A band of 13 kDa was observed by western blotting (23).

## Conclusion

*ROM4* gene was successfully cloned into pTG19-T vector and then into pcDNA3 expression vector for protein expression. The used eukaryotic expression system is an appropriate system for high-level recombinant protein production of *ROM4* gene from *T. gondii* tachyzoites used as antigenic component for vaccine development and serological assay.

## References

[1] Tenter AM Heckeroth AR Weiss LM *Toxoplasma gondii*: from animals to humans. Int J Parasitol. 2000; 30(12–13:1217–58. 11113252 10.1016/s0020-7519(00)00124-7 PMC3109627

[2] Rahimi MT Mahdavi SA Javadian B High seroprevalence of *Toxoplasma gondii* antibody in HIV/AIDS individuals from North of Iran. Iran J Parasitol. 2015; 10(4):584–9. 26811725 PMC4724835

[3] Sarvi S Daryani A Rahimi MT Cattle toxoplasmosis in Iran: a systematic review and meta–analysis. Asian Pac J Trop Med. 2015; 8(2):120–6. 25902025 10.1016/S1995-7645(14)60301-1

[4] Kazemi B Bandehpour M Maghen L Gene cloning of 30 kDa *Toxoplasma gondii* tachyzoites surface antigen (SAG1). Iran J Parasitol. 2007; 2(2): 1–8.

[5] Dubey JP Jones JL *Toxoplasma gondii* infection in humans and animals in the United States. Int J Parasitol. 2008; 38(11):1257–78. 18508057 10.1016/j.ijpara.2008.03.007

[6] Tekkesin N Diagnosis of toxoplasmosis in pregnancy: a review. HOAJ Biol. 2012; 1(1): 9–12.

[7] Rahimi MT Daryani A Sarvi S Cats and *Toxoplasma gondii*: A systematic review and meta-analysis in Iran. Onderstepoort J Vet Res. 2015;82(1):823. 26017063 10.4102/ojvr.v82i1.823 PMC6238687

[8] Chen J Li ZY Petersen E DNA vaccination with genes encoding *Toxoplasma gondii* antigens ROP5 and GRA15 induces protective immunity against toxoplasmosis in Kunming mice. Expert Rev Vaccines. 2015;14(4):617–24. 25749394 10.1586/14760584.2015.1011133

[9] Santos JM Ferguson DJ Blackman MJ Intra membrane cleavage of AMA1 triggers *Toxoplasma* to switch from an invasive to a replicative mode. Science. 2011;331(6016):473–7. 21205639 10.1126/science.1199284

[10] Zhang NZ Xu Y Wang M Protective efficacy of two novel DNA vaccines expressing *Toxoplasma gondii* rhomboid 4 and rhomboid 5 proteins against acute and chronic toxoplasmosis in mice. Expert Rev Vaccines. 2015;14 (9):1289–97. 26111968 10.1586/14760584.2015.1061938

[11] Abdi J Kazemi B Mohebali M Gene cloning, expression and serological evaluation of the 12-kDa antigen-B subunit from *Echinococcus granulosus*. Ann Trop Med Parasitol. 2010; 104(5):399–407. 20819308 10.1179/136485910X12743554760261

[12] Zhang NZ Chen J Wang M Vaccines against *Toxoplasma gondii*: new developments and perspectives. Expert Rev Vaccines. 2013;12(11):1287–99. 24093877 10.1586/14760584.2013.844652

[13] Buxton D Thomson K Maley S Vaccination of sheep with a live incomplete strain (S48) of *Toxoplasma gondii* and their immunity to challenge when pregnant. Vet Rec. 1991;129(5):89–93. 1926725 10.1136/vr.129.5.89

[14] Dowse TJ Pascall JC Brown KD Apicomplexan rhomboids have a potential role in microneme protein cleavage during host cell invasion. Int J Parasitol. 2005;35(7):747–56. 15913633 10.1016/j.ijpara.2005.04.001

[15] Sibley LD The roles of intramembrane proteases in protozoan parasites. Biochim Biophys Acta. 2013;1828(12):2908–15. 24099008 10.1016/j.bbamem.2013.04.017 PMC3793208

[16] Brossier F Jewett TJ Sibley LD A spatially localized rhomboid protease cleaves cell surface adhesins essential for invasion by *Toxoplasma*. Proc Natl Acad Sci U S A. 2005; 102(11:4146–51. 15753289 10.1073/pnas.0407918102 PMC554800

[17] Li J Han Q Gong P *Toxoplasma* *gondii* rhomboid protein 1 (TgROM1) is a potential vaccine candidate against toxoplasmosis. Vet Parasitol. 2012:184(2–4):154–60. 21906881 10.1016/j.vetpar.2011.08.014

[18] Shen B Buguliskis JS Lee TD Functional analysis of rhomboid proteases during *Toxoplasma* invasion. MBio. 2014; 5(5):e01795–14. 25336455 10.1128/mBio.01795-14 PMC4212836

[19] Huynh MH Carruthers VB *Toxoplasma* MIC2 is a major determinant of invasion and virulence. PLoS Pathog. 2006;2(8):e84. 16933991 10.1371/journal.ppat.0020084 PMC1550269

[20] Yang HL Xiao JH Liang Y Cloning, sequencing and expressing of microneme protein 1 partial gene in *Toxoplasma gondii* ZS2 isolate. Zhonghua Yu Fang Yi Xue Za Zhi. 2003;37(1):29–32. 12760792

[21] Artama WT Dewi NNA Subekti DT Cloning gene encoding micronema 3 (Mic3) protein of tachyzoite *Toxoplasma gondii* local isolate. Indonesian J Biotechnol. 2011; 10(1): 5–9.

[22] Babaie J Zare M Sadeghiani G Bacterial production of dense granule antigen GRA8 of *Toxoplasma gondii*. Iran Biomed J. 2009:13(3):145–51. 19688020

[23] Naserifar R Ghaffarifar F Dalimi Cloning of *Toxoplasma Gondii* Granular Antigen 5(GRA5) in Expression Eukaryotic Plasmid pcDNA3 and its Expression on CHO Cell. J Ilam Uni Med SCI. 2011; 19 (3: 1–12.

